# Associations Among Outdoor Time, Skin Tanning, and the Risk of Surgically Treated Cataract for Australians 45 to 65 Years of Age

**DOI:** 10.1167/tvst.11.6.3

**Published:** 2022-06-02

**Authors:** Xiaotong Han, Jiaqing Zhang, Wei Wang, Zhenzhen Liu, Xuhua Tan, Xianwen Shang, Lei Zhang, Mingguang He, Lixia Luo

**Affiliations:** 1 State Key Laboratory of Ophthalmology, Zhongshan Ophthalmic Center, Sun Yat-sen University, Guangdong Provincial Key Laboratory of Ophthalmology and Visual Science, Guangdong Provincial Clinical Research Center for Ocular Diseases, Guangzhou, China; 2 Centre for Eye Research Australia, Royal Victorian Eye and Ear Hospital, Melbourne, Victoria, Australia; 3 Melbourne Sexual Health Centre, Alfred Health, Melbourne, Victoria, Australia; 4 Department of Epidemiology and Biostatistics, College of Public Health, Zhengzhou University, Zhengzhou, Henan, China; 5 Ophthalmology, Department of Surgery, University of Melbourne, Melbourne, Victoria, Australia

**Keywords:** cataract, outdoor, skin tanning, cataract surgery, sun exposure

## Abstract

**Purpose:**

To investigate the association between outdoor time and the risk of cataract surgery in a large Australian population.

**Methods:**

This was a population-based prospective cohort study with 137,133 participants 45 to 65 years of age and without prior history of cataract surgery from the 45 and Up Study. Outdoor hours per day on weekdays and weekends, as well as tanning with repeated sun exposure, were assessed by a self-administered baseline questionnaire. Cataract surgery events were confirmed by the Medicare Benefits Schedule from baseline until the end of follow-up in 2016.

**Results:**

During a mean follow-up of 9 years, 14,338 participants received cataract surgery with a corresponding incidence of 10.5%. Multiple Cox regression analysis showed that more outdoor hours on weekends (*P* trend < 0.001) and the ability to get tanned by repeated sun exposure (*P* trend = 0.041) were significantly associated with a lower risk of cataract surgery, whereas more outdoor hours on weekdays were nominally significantly associated (*P* trend = 0.055). Participants who spent 10+ hours outdoors on weekends had 9% decreased risk compared with those who spent ≤2 hours outdoors. In addition, compared to participants who got very tanned by repeated sun exposure, those less likely to get tanned had a 5% to 7% increased risk of cataract surgery.

**Conclusions:**

In this large Australian cohort 45 to 65 years of age, more outdoor time and ease of tanning with sun exposure were associated with a lower incidence of cataract surgery.

**Translational Relevance:**

With proper sun protection, more outdoor time may lead to a lower risk of severe cataracts requiring surgery.

## Introduction

Cataract is the leading cause of global blindness, and the related burden is projected to increase substantially with population growth, aging, and extended longevity.^1^^,^^2^ In addition to the direct detrimental effects of cataract on vision, the association between cataract and a reduced quality of life, as well as increased morbidity and mortality, have been widely reported.^3^^,^^4^ A complex interplay of sociodemographic, environmental, and genetic factors underlies the pathogenesis of cataract development and progression. Acquiring a better understanding of modifiable risk factors in daily life would aid in identifying effective precautions to lower individual risk.

Many risk factors have been widely reported for cataract, including older age, diabetes mellitus, and steroid use.^5^^,^^6^ Ultraviolet (UV) radiation, which affects the human eye in everyday life, has been consistently reported as a risk factor for cataract.^7^^,^^8^ However, the detrimental effect of sun exposure in our daily life on cataract has been reported to be modest and mostly pertains to cortical cataract. Whether UV radiation can induce more severe forms of cataract (e.g., requiring surgical treatment) has been less investigated.^7^^,^^9^^,^^10^ We think the potential severity of cataract is of great importance, as people would tend not to neglect the clear benefits of outdoor activities with regard to our overall mental and physical well-being when faced with only a slight increase in lens opacity.

Australia is often referred to as a sun-blessed country that offers residents rich relationships with the outdoors.^11^ Australians receive high levels of UV radiation, making them an ideal population for assessing sun exposure–related health outcomes.^12^^,^^13^ Therefore, this study aimed to investigate the associations among outdoor time, skin tanning, and the risk of cataract requiring surgical treatment over a 10-year study period based on the Sax Institute's 45 and Up Study, which is the largest population-based prospective cohort study in Australia.^14^

## Methods

### Participants

Participants from The Sax Institute's 45 and Up Study 45 years of age and older living in New South Wales (NSW), an area of high ambient solar UV in Australia, were randomly sampled from the general population using the Services Australia (formerly the Australian Government Department of Human Services) Medicare enrollment database.^14^ A total of 267,153 participants were recruited from a baseline from 2006 to 2009, corresponding to 11% of the entire NSW population of this age group. A study invitation, a self-administered questionnaire, and a consent form were mailed to each participant at baseline, and these documents were mailed back to the study coordinating center when completed. All participants provided signed consent for their data to be linked to a range of Australian health databases, including the Medicare Benefits Schedule (MBS), which tracked claims records for diagnostic tests and procedures from January 24, 2001, to December 31, 2016. Detailed study methods have been described in earlier studies.^14^^,^^15^ The conduct of the 45 and Up Study was approved by the University of New South Wales Human Research Ethics Committee. The study protocol was approved by the Royal Victorian Eye and Ear Hospital Human Research Ethics Committee (17/1330HS/20).

This study included only participants 45 to 65 years old because they were more physically active and subjects 65 years or older were highly likely to have already received cataract surgery at baseline. Participants with a history of cataract surgery based on the baseline questionnaire or MBS database were excluded from the study. Participants with a physical function score of less than 25 were deemed as having physical restrictions and also excluded from the study. Other exclusion criteria included history of juvenile cataract extraction, history of vitrectomy or ocular traumatic surgeries, and missing data for outdoor time or skin tanning. The participant selection process is shown in the Figure.

**Figure.**
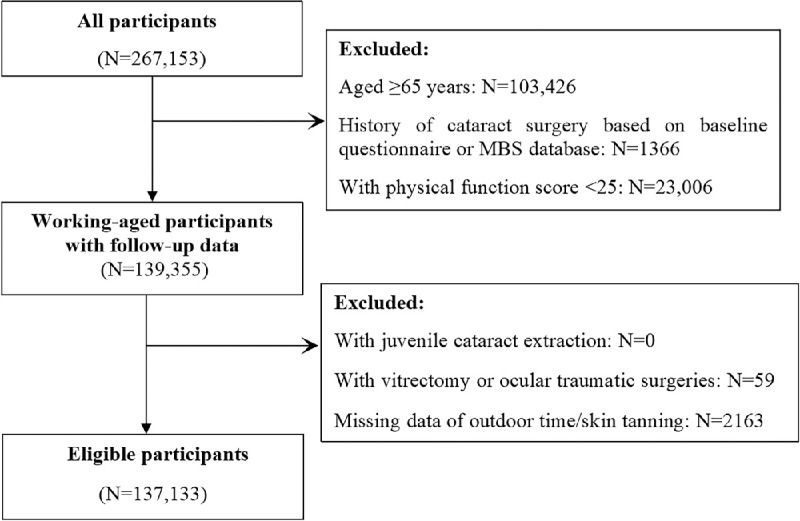
Participant selection flowchart.

### Measurements

The baseline questionnaire collects information on a broad range of demographic, socioeconomic, medical and lifestyle factors and is available on the website of the 45 and Up Study (http://www.saxinstitute.org.au/our-work/45-up-study/questionnaires/). Demographic (including age, sex, education, height, weight), medical factors (including history of hypertension and diabetes), and lifestyle factors (including physical activity [PA] and drinking and smoking status) were collected from the baseline questionnaire. Age was categorized into four groups of 45 to 49, 50 to 54, 55 to 59, and 60 to 65 years of age; household income was also categorized into four groups of <20,000, 20,000 to 40,000, 40,000 to 70,000, and >70,000 Australian dollars (AUD)/year. Educational attainment was determined by the highest qualification participants had completed; the options included no school certificate or other qualifications amounting to less than 10 years of school, high school or trade, or a university degree or higher. Smoking status assessment has been reported in detail in previous studies, and current and past smokers were both deemed as smokers in the current study. Alcohol intake was defined as the number of alcohol drinks per week based on the question, “About how many alcoholic drinks do you have each week?” Participants were dichotomized into <14 drinks and ≥14 drinks per week accordingly. We calculated the metabolic equivalent intensity level for the number of sessions of PA per week and categorized them into four groups of <5, ≥5 to 9, ≥9 to 14, and ≥14 sessions per week. Detailed assessment and calculation methods for PA have been reported previously.^16^

Outdoor time was assessed based on multiple-choice questions from the baseline questionnaire (e.g., “About how many hours a day would you usually spend outdoors on a weekday and on the weekend?”). Skin tanning was based on the question “What would happen if your skin was repeated exposed to bright sunlight during summer without any protection?” Outdoor hours per day on weekdays and weekends were summarized separately in the questionnaire database and categorized into four groups: ≤2, 3 to 4, 5 to 10, and >10 hours per day. Tanning with repeated sun exposure was also categorized into four groups in the questionnaire database: get very tanned, get moderately tanned, get mildly or occasionally tanned, and never tan or only get freckled. In addition, the color on the inside of upper arm was assessed by the question “What best describes the color of the skin on the inside of your upper arm—that is, your skin color without any tanning?” Six options were provided, including very fair, fair, light olive, dark olive, brown, and black.

### Outcome Definition

The primary outcome was occurrence of cataract surgery during the follow-up. Two procedures codes (42698, Lens Extraction, and 42702, Lens Extraction and Insertion of Intraocular Lens) represent cataract surgery in the MBS and were used to differentiate participants who did and did not receive cataract surgery in at least one eye during the follow-up period.

### Statistical Analysis

All data analysis was performed using SAS 9.4 (SAS Institute, Cary, NC). Body mass index (BMI) was calculated as weight divided by the square of height and further categorized into three groups based on the World Health Organization criteria: normal (<25 kg/m^2^), overweight (25.0–29.99 kg/m^2^), and obese (30–50 kg/m^2^). Descriptive statistics, including frequencies and proportions, were used to characterize the study population at baseline. The χ^2^ test was used to compare baseline characteristics of the participants with and without claim records of cataract surgery during the follow-up. Two Cox regression models were used to evaluate the association of outdoor hours per day on weekdays and weekends and tanning with repeated sun exposure with the risk of cataract surgery. Model 1 was adjusted for demographic and medical factors, and model 2 was additionally adjusted for lifestyle factors and color of skin of the inside of the upper arm. *P* < 0.05 was considered to be of statistical significance.

## Results

Among the 267,153 participants recruited at baseline (2006–2009), 137,133 participants (51.3%) were finally included in the current analysis (Fig.). During the mean 9-year follow-up period (range, 7.0–11.5 years), 14,338 eligible participants (11.4%) received cataract surgery in at least one eye, corresponding to an incidence of 10.5%.

Participants’ baseline characteristics are shown in Table 1. Compared to participants who did not receive cataract surgery during the follow-up, those who did were more likely to be of older age (P < 0.001), to be female (P < 0.001), to have a history of hypertension (P < 0.001) or diabetes (P < 0.001), to consume fewer than 14 alcoholic drinks per week (P = 0.003), and to be a non-smoker (P < 0.001). The two groups of participants did not differ significantly in household income, education level, BMI, or PA level. However, the distribution of time outdoors on weekdays (P = 0.001) and on weekends (P < 0.001) and tanning with repeated sun exposure (P < 0.001) were significantly different between the two groups.

**Table 1.** Characteristics of Participants Who Did and Did Not Undergo Cataract Surgery During the Follow-Up

		Cataract Surgery, *n* (%)		
Factor	All, *n* (%)	Yes (*n* = 14,338)	No (*n* = 122,795)	*P*
Baseline age (y)				<0.001
45–49	30,977 (22.6)	929 (6.5)	30,048 (24.5)	
50–54	37,072 (27.0)	2187 (15.3)	34,885 (28.4)	
55–59	37,695 (27.5)	4452 (31.1)	33,243 (27.1)	
60–65	31,389 (22.9)	6770 (47.2)	24,619 (20.0)	
Sex				<0.001
Male	60,312 (44.0)	5773 (40.3)	54,539 (44.4)	
Female	76,821 (56.0)	8565 (59.7)	68,256 (55.6)	
Household income (AUD/y)				0.653
<20,000	11,244 (8.2)	1059 (7.4)	10,185 (8.3)	
20,000–40,000	19,249 (14.0)	2201 (15.4)	17,048 (13.9)	
40,000–70,000	30,966 (22.6)	3413 (23.8)	27,553 (22.4)	
>70,000	51,556 (37.6)	4822 (33.6)	46,734 (38.1)	
Missing	24,118 (17.6)	2843 (19.8)	21,275 (17.3)	
Highest education				0.556
University	9381 (6.8)	883 (6.2)	8498 (6.9)	
High school/technical and further education (TAFE)	84,902 (61.9)	9013 (62.9)	75,889 (61.8)	
<10 y	41,646 (30.4)	4313 (30.1)	37,333 (30.4)	
BMI				0.502
Normal	50,040 (36.5)	5133 (35.8)	44,907 (36.6)	
Overweight	51,152 (37.3)	5472 (38.2)	45,680 (37.2)	
Obesity	28,244 (20.6)	2994 (20.9)	25,250 (20.6)	
Missing	7697 (5.6)	739 (5.2)	6958 (5.7)	
Diabetes				<0.001
Yes	6844 (5.0)	1123 (7.8)	5721 (4.7)	
No	130,289 (95.0)	13,215 (92.2)	117,074 (95.3)	
Hypertension				<0.001
Yes	35,135 (25.6)	4640 (32.4)	30,495 (24.8)	
No	101,998 (74.4)	9698 (67.6)	92,300 (75.2)	
Smoker (current + past)				<0.001
Yes	11,528 (8.4)	780 (5.4)	10,748 (8.8)	
No	125,600 (91.6)	13,558 (94.6)	112,042 (91.2)	
Alcohol consumption per week				0.003
<14 drinks	98,569 (71.9)	10,463 (73.0)	88,106 (71.8)	
≥14 drinks	36,865 (26.9)	3678 (25.7)	33,187 (27.0)	
Physical activity (sessions/wk)				0.140
<5	22,279 (16.2)	2166 (15.1)	20,113 (16.4)	
≥5–9	39,680 (28.9)	4217 (29.4)	35,463 (28.9)	
≥9–14	32,985 (24.1)	3601 (25.1)	29,384 (23.9)	
≥14	39,027 (28.5)	4017 (28.0)	35,010 (28.5)	
Tanning with repeated sun exposure				<0.001
Get very tanned	41,168 (30.0)	3971 (27.7)	37,197 (30.3)	
Get moderately tanned	55,048 (40.1)	5897 (41.1)	49,151 (40.0)	
Get mildly or occasionally tanned	29,595 (21.6)	3210 (22.4)	26,385 (21.5)	
Never tan only get freckled	11,322 (8.3)	1260 (8.8)	10,062 (8.2)	
Outdoors on weekdays (hr/d)				0.007
≤2	79,635 (58.1)	8042 (56.1)	71,593 (58.3)	
3–4	24,582 (17.9)	2949 (20.6)	21,633 (17.6)	
5–10	19,015 (13.9)	2065 (14.4)	16,950 (13.8)	
10+	11,122 (8.1)	1000 (7.0)	10,122 (8.2)	
Outdoors on weekends (hr/d)				<0.001
≤2	30,517 (22.3)	3585 (25.0)	26,932 (21.9)	
3–4	41,500 (30.3)	4422 (30.8)	37,078 (30.2)	
5–10	45,930 (33.5)	4581 (32.0)	41,349 (33.7)	
10+	16,104 (11.7)	1427 (10.0)	14,677 (12.0)	

Cox regression analysis showed that, after adjusting for multiple confounders including age, sex, income, ethnicity, BMI, diabetes, hypertension, smoking, drinking, PA, and color of skin on the inside of the upper arm, fewer outdoor hours on weekends (P trend < 0.001) and milder tanning with repeated sun exposure (P trend = 0.041) were significantly associated with a higher risk of cataract surgery during the follow-up (Table 2). Compared to participants who spent less than 2 hours outdoor on weekend, those who spent more than 10 hours had a 9% decreased risk of cataract surgery. Compared to participants who get very tanned with repeated sun exposure, those who never tan or only get freckled had a 5% increased risk of cataract surgery. In addition, participants who spent more than 10 hours outdoors on weekdays also had a significantly lower risk of cataract surgery compared to those who spent less than 2 hours (hazard ratio [HR] = 0.93; 95% confidence interval [CI], 0.86–0.99), although the trend analysis was not statistically significant (P = 0.055).

**Table 2.** Cox Regression Analysis of Associations Between Sun Exposure and the Risk of Cataract Surgery During the Follow-Up

		Univariate Analysis		Multiple Regression Analysis			
				Model 1		Model 2	
Factors	Events/Participants, *n* (%)	HR (95% CI)	*P* for Trend	HR (95% CI)	*P* for Trend	HR (95% CI)	*P* for Trend
Outdoors on weekday (hr/d)							
≤2 (ref.)	8042/79,635 (10.1)	1.0	0.537	1.0	0.035	1.0	0.055
3–4	2949/24,582 (12)	1.20 (1.15–1.25)	—	1.01 (0.96–1.05)	—	1.01 (0.97–1.06)	—
5–10	2065/19,015 (10.86)	1.08 (1.03–1.14)	—	0.97 (0.92–1.02)	—	0.98 (0.93–1.03)	—
10+	1000/11,122 (8.99)	0.89 (0.84–0.95)	—	0.92 (0.86–0.99)	—	0.93 (0.86–0.99)	—
Outdoors on weekend (hr/d)							
≤2 (ref.)	3585/30,517 (11.75)	1.0	<0.001	1.0	<0.001	1.0	<0.001
3–4	4422/41,500 (10.66)	0.91 (0.87–0.95)	—	0.94 (0.90–0.99)	—	0.94 (0.90–0.99)	—
5–10	4581/45,930 (9.97)	0.85 (0.82–0.89)	—	0.92 (0.88–0.97)	—	0.92 (0.88–0.97)	—
10+	1427/16,104 (8.86)	0.76 (0.71–0.81)	—	0.91 (0.85–0.97)	—	0.91 (0.85–0.97)	—
Tanning with repeated sun exposure							
Get very tanned (ref.)	3971/41,168 (9.65)	1.0	<0.001	1.0	0.048	1.0	0.041
Get moderately tanned	5897/55,048 (10.71)	1.12 (1.08–1.17)	—	1.03 (0.99–1.08)	—	1.05 (1.00–1.09)	—
Get mildly or occasionally tanned	3210/29,595 (10.85)	1.15 (1.09–1.20)	—	1.05 (1.00–1.10)	—	1.07 (1.01–1.13)	—
Never tan, only get freckled	1260/11,322 (11.13)	1.17 (1.10–1.25)	—	1.04 (0.98–1.11)	—	1.05 (0.97–1.13)	—

Model 1 was adjusted for age, sex, income, education, ethnicity, BMI, diabetes, and hypertension. Model 2 was further adjusted for smoker, alcohol, physical activity, and color of skin on the inside of the upper arm.

Subgroup analysis showed that the interactive associations between cataract surgical risk and outdoor hours on weekdays and weekend were not influenced by age, gender, BMI, smoking, drinking, or PA status (Tables 3, 4). A significant trend toward a larger reduction of cataract surgical risk with longer outdoor times on weekdays was observed among the following subgroup of participants: 60 to 65 years of age (P for trend <0.001), men (P for trend = 0.002), women (P for trend = 0.005), overweight (P for trend = 0.009), and obese (P for trend = 0.001). A similar trend was also observed for participants with varying smoking, alcohol consumption, and PA status (except for PA > 5 to 9 sessions per week). Regarding time spent outdoors on weekends, a significant trend in reduced cataract surgical risk with longer time spent outdoors was observed among participants 60 to 65 years of age (P for trend = 0.002), women (P for trend < 0.001), overweight participants (P for trend = 0.004), non-smokers (P for trend = 0.001), and participants with varying alcohol drinking status and with PA levels of <5 sessions per week (P for trend = 0.001) and >9 to 14 sessions/week (P = 0.008). The increased cataract surgical risk related to milder tanning with repeated sun exposure was more pronounced among older participants (P for interaction = 0.003), whereas interactions with other factors were not significant (Table 5). Participants 65 to 65 years of age who never tanned or only get freckled had an 11% increased risk of cataract surgery compared to those who get very tanned. It should be noted that consideration should be given to the generalizability of the results and conclusions, given the participation rate of ∼18%.

**Table 3.** Subgroup Analysis of Associations Between Weekday Outdoor Hours Per Day and the Risk of Cataract Surgery During the Follow-Up

	Weekday Outdoor Hours Per Day				
	3–4 vs. ≤2	5–10 vs. ≤2	10+ vs. ≤2		*P* for
Factors	HR (95% CI)	HR (95% CI)	HR (95% CI)	*P* for Trend	Interaction
Baseline age (y)					0.303
45–49	0.95 (0.78–1.16)	1.01 (0.81–1.26)	0.84 (0.63–1.12)	0.208	
50–54	0.94 (0.82–1.06)	0.96 (0.82–1.11)	1.06 (0.89–1.27)	0.266	
55–59	1.04 (0.96–1.13)	0.99 (0.90–1.09)	0.95 (0.84–1.08)	0.096	
60–65	1.01 (0.95–1.08)	0.97 (0.90–1.04)	0.88 (0.80–0.98)^a^	<0.001	
Sex					0.640
Male	1.01 (0.94–1.08)	0.98 (0.91–1.05)	0.93 (0.85–1.02)	0.002	
Female	1.01 (0.96–1.07)	0.98 (0.91–1.05)	0.92 (0.83–1.03)	0.005	
BMI					0.504
Normal	1.02 (0.95–1.10)	1.01 (0.93–1.10)	0.93 (0.82–1.06)	0.080	
Overweight	0.98 (0.91–1.05)	0.94 (0.86–1.02)	0.96 (0.86–1.07)	0.009	
Obesity	1.02 (0.93–1.13)	0.93 (0.83–1.05)	0.85 (0.73–0.98)^a^	0.001	
Smoker (current + past)					0.019
Yes	0.88 (0.73–1.06)	0.89 (0.73–1.10)	0.72 (0.55–0.94)^a^	<0.001	
No	1.02 (0.97–1.07)	0.98 (0.93–1.03)	0.94 (0.88–1.01)	<0.001	
Alcohol consumption per week					0.987
<14 drinks	1.02 (0.97–1.07)	0.99 (0.94–1.05)	0.92 (0.85–1.00)	0.002	
≥14 drinks	0.98 (0.90–1.07)	0.90 (0.81–1.00)	0.94 (0.82–1.08)	<0.001	
Physical activity (sessions/wk)					0.716
<5	0.93 (0.82–1.06)	0.84 (0.73–0.98)^a^	0.94 (0.78–1.12)	0.014	
>5–9	1.00 (0.92–1.09)	1.05 (0.95–1.16)	0.99 (0.87–1.13)	0.387	
>9–14	1.02 (0.94–1.11)	0.92 (0.83–1.02)	0.87 (0.75–1.01)	0.002	
>14	1.04 (0.96–1.13)	1.02 (0.93–1.11)	0.92 (0.81–1.03)	0.019	

a Indicates *P* value < 0.05.

**Table 4.** Subgroup Analysis of Associations Between Weekend Outdoor Hours Per Day and the Risk of Cataract Surgery During the Follow-Up

	Weekend Outdoor Hours Per Day				
	3–4 vs. ≤2	5–10 vs. ≤2	10+ vs. ≤2		*P* for
Factors	HR (95% CI)	HR (95% CI)	HR (95% CI)	*P* for Trend	Interaction
Baseline age (y)					0.293
45–49	0.96 (0.80–1.16)	0.97 (0.81–1.17)	0.90 (0.71–1.16)	0.486	
50–54	0.91 (0.82–1.03)	0.94 (0.83–1.05)	0.89 (0.76–1.05)	0.185	
55–59	0.92 (0.85–1.00)	0.92 (0.84–1.00)	0.96 (0.85–1.07)	0.153	
60–65	0.97 (0.91–1.03)	0.92 (0.86–0.99)^a^	0.90 (0.81–0.99)^a^	0.002	
Sex					0.107
Male	0.97 (0.89–1.06)	0.95 (0.88–1.03)	0.95 (0.86–1.05)	0.217	
Female	0.94 (0.89–0.99)^a^	0.92 (0.87–0.97)^a^	0.87 (0.79–0.96)^a^	<0.001	
BMI					0.233
Normal	0.91 (0.85–0.98)^a^	0.93 (0.86–1.00)^a^	0.92 (0.82–1.03)	0.053	
Overweight	0.98 (0.91–1.06)	0.90 (0.83–0.97)^a^	0.90 (0.81–1.00)^a^	0.004	
Obesity	0.94 (0.85–1.04)	0.94 (0.85–1.05)	0.90 (0.79–1.03)	0.116	
Smoker (current + past)					0.939
Yes	1.09 (0.88–1.34)	0.92 (0.74–1.13)	0.83 (0.64–1.08)	0.107	
No	0.94 (0.90–0.98)^a^	0.92 (0.88–0.97)^a^	0.92 (0.86–0.98)^a^	0.001	
Alcohol consumption per week					0.110
<14 drinks	0.96 (0.91–1.01)	0.93 (0.88–0.99)^a^	0.93 (0.86–1.00)	0.008	
≥14 drinks	0.92 (0.84–1.00)	0.91 (0.83–0.99)^a^	0.88 (0.77–1.01)	0.003	
Physical activity (sessions/wk)					0.724
<5	0.83 (0.75–0.93)^a^	0.82 (0.73–0.92)^a^	0.85 (0.72–1.00)	0.002	
>5–9	0.97 (0.89–1.05)	1.00 (0.92–1.08)	0.95 (0.84–1.07)	0.597	
>9–14	0.94 (0.86–1.03)	0.89 (0.81–0.97)^a^	0.89 (0.78–1.02)	0.008	
>14	1.01 (0.92–1.10)	0.96 (0.88–1.05)	0.94 (0.84–1.06)	0.115	

a Indicates *P* value < 0.05.

**Table 5.** Subgroup Analysis of Associations Between Tanning With Repeated Sun Exposure and the Risk of Cataract Surgery During the Follow-Up

	Weekend Outdoor Hours Per Day				
	Moderately vs. Very	Mildly vs. Very	Never vs. Very		*P* for
Factors	Tanned HR (95% CI)	Tanned HR (95% CI)	Tanned HR (95% CI)	*P* for Trend	Interaction
Baseline age (y)					0.003
45–49	0.90 (0.77–1.06)	0.89 (0.73–1.09)	0.74 (0.55–1.00)	0.165	
50–54	1.04 (0.94–1.16)	1.03 (0.90–1.18)	0.93 (0.77–1.14)	0.305	
55–59	1.09 (1.01–1.18)^a^	1.11 (1.01–1.21)^a^	1.07 (0.93–1.22)	0.194	
60–65	1.04 (0.98–1.11)	1.08 (1.00–1.16)^a^	1.11 (1.00–1.23)^a^	0.008	
Sex					0.376
Male	1.07 (1.00–1.14)^a^	1.12 (1.03–1.21)^a^	1.04 (0.91–1.18)	0.075	
Female	1.02 (0.96–1.08)	1.03 (0.96–1.11)	1.03 (0.94–1.13)	0.296	
BMI					0.857
Normal	1.05 (0.98–1.13)	1.11 (1.02–1.21)^a^	1.09 (0.96–1.23)	0.127	
Overweight	1.01 (0.95–1.09)	1.04 (0.95–1.13)	1.02 (0.90–1.15)	0.384	
Obesity	1.07 (0.98–1.18)	1.06 (0.94–1.18)	1.04 (0.89–1.21)	0.511	
Smoker (current + past)					0.225
Yes	0.98 (0.83–1.17)	1.06 (0.86–1.32)	0.85 (0.60–1.19)	0.921	
No	1.05 (1.00–1.10)^a^	1.07 (1.01–1.13)^a^	1.06 (0.98–1.14)	0.056	
Alcohol consumption per week					0.679
<14 drinks	1.04 (0.99–1.09)	1.07 (1.00–1.13)^a^	1.07 (0.98–1.17)	0.086	
≥14 drinks	1.06 (0.97–1.15)	1.06 (0.96–1.17)	1.01 (0.89–1.16)	0.311	
Physical activity (sessions/wk)					0.842
<5	1.00 (0.90–1.12)	1.08 (0.95–1.23)	1.07 (0.90–1.27)	0.209	
≥5–9	1.02 (0.94–1.11)	1.05 (0.96–1.16)	1.05 (0.92–1.19)	0.361	
≥9–14	1.06 (0.97–1.15)	1.04 (0.94–1.16)	0.97 (0.83–1.12)	0.979	
≥14	1.08 (1.00–1.17)^a^	1.10 (0.99–1.21)	1.11 (0.96–1.27)	0.097	

a Indicates *P* value < 0.05.

## Discussion

Cataract formation and progression are variously affected by many factors. The association between outdoor sun exposure and cataract risk should be investigated in a large sample with appropriate control of the many confounding factors. Many previous studies have consistently reported positive associations between increased sun exposure and cataract, but most have had small sample sizes and failed to account for confounders; their case-control or cross-sectional study design has also hindered assessment of causal associations.^10^ One case-control study (354 participants) in Australia reported strong positive associations between occupational sun exposure and nuclear cataract,^7^ whereas another case-control study (677 participants) in Spain found no significant associations between years of outdoor exposure and cataract risk.^17^ The Taizhou Eye Study, based on 2006 rural Chinese adults, reported outdoor activity as a risk factor for cortical cataract with an odds ratio of 1.043, whereas another study of Han Chinese people living in areas with varying UV intensities reported that high UV exposure was associated with an increased odds ratio for nuclear cataract.^8^^,^^9^ The CASA study of geographically diverse populations of India included 9735 participants and found that nuclear and cortical cataracts showed a positive association with increased sun exposure but not posterior subcapsular cataract.^10^ The U.S. Radiologic Technologist Study reported that ambient UV radiation was associated with increased risk of cataract and cataract surgery.^18^

Although it is true that UV exposure has a detrimental effect on human lenses, existing evidence from previous studies indicates only a modest increase in overall cataract risk with outdoor sun exposure.^19^^,^^20^ However, we should primarily be concerned with the risk of severe cataract, as the benefits of sun exposure and outdoor activity should not be overlooked, especially in light of the potential for mild cloudiness of the lens having a negligible effect on vision and quality of life. Our study included 137,133 participants from a representative population-based Australian cohort and found that increased outdoor time was associated with a lower risk of cataract requiring surgical treatment during a 10-year follow-up. To our knowledge, this is the largest cohort study with the longest follow-up period regarding this issue, and 12 potential confounding factors were adjusted for in the regression model. One might argue that sun protection behaviors, including wearing sunglasses, could directly influence the risk of cataract but these were not adjusted for in the regression model due to a lack of data regarding this variable. In our study, we assumed that the sunglasses wearing rate was similar for people who did and did not receive cataract surgery, given that people in these two groups had similar education level and income status (Table 1). Considering the older age in the cataract surgery group and the significant benefit of time spent outdoors with regard to cataract surgery risk in older participants (Tables 1, 3), it is unlikely that further adjusting for sunglasses wearing behavior would change the study findings. It has been reported that 71% of Australians own at least one pair of sunglasses, and 79% of them wear them most of the time when outside on a sunny day.^21^ Thus, our study findings might not be directly applied to other regions with lower rates of sunglass wear. Nevertheless, we clearly recommend that people wear sunglasses outdoors to minimize the detrimental effects of direct UV light on lens. In a response letter published in the *British Medical Journal*, the author suggested that sunlight was not responsible for cataracts unless co-affected by other factors and that people should go out in the sun more, not less.^22^ The effect of UV exposure on lenses has been suggested to be related to oxidative stress,^23^ but the underlying mechanism of the protective effect of outdoor time on surgically treated cataract in this study is unclear, which could be related to a higher antioxidative capability with more outdoor activities or increased vitamin D level. More studies are needed in the future to better understand the association.

Another interesting finding of the current study is that people who were more likely to get tanned with repeated sun exposure had lower risk of cataract surgery during the follow-up compared to those less likely to get tanned, even after adjusting for ethnicity. Tanning of the skin depends primarily on the degree of UV radiation and the amount of melanin in the skin.^24^ A suntan is a sign that the skin is releasing melanin, which is a natural protector against the UV rays of the sun.^25^ Given that epidermal melanin varies among ethnicities, the study finding was adjusted for the color of the skin on the inside of the upper arm. Easy tanning with sun exposure was believed to be the most important photoprotective factor, considering that melanin has both broadband UV absorbent and antioxidant properties. Previous studies have also found that subjects with darker skin or who easily suntan had a lower incidence of skin cancer.^26^ We suggest that one explanation could be that those who tan easily have higher melanin levels and better antioxidant capability and thus a lower risk of cataract. Older participants who tanned less were found to have increased cataract risk, which could be due to reduced melanin levels due to older age.^27^ The regulatory mechanisms involved in skin pigmentation are complex and not fully understood, so future studies are necessary to further illustrate the underlying biological associations.

This strengths of this study lie in its population-based prospective cohort design, its large sample size, the availability of a comprehensive set of variables, and its long-term follow-up. However, several limitations of this study should be noted, as well. First, despite the large sample size that provided adequate power, cataract surgeries performed in public hospitals were not taken into account in the current study, given the difficulty of gathering data for such a large population. It would be nearly impossible to collect complete information for such a large population. It has been reported that 72% of the cataract surgeries during the study period were performed in private facilities, suggesting a good representativeness of our study sample.^28^ Second, direct UVA/UVB measurements reflecting the intensity and extent of sun exposure were not available for this study. Also, data for sun-exposure behavior were only available at baseline, and the current analyses were based on the assumption that these adult participants would not change their habits during the follow-up. We had no data from follow-up questionnaires, so potential behavior changes during the follow-up were not assessed. Third, data regarding the use of sunglasses and hats were not available for this study and were not adjusted for in the statistical model. This might bias the study findings but to what extent we did not know. Finally, gene polymorphisms may be confounders in the relationship of cataract with UV radiation exposure, but this was not accounted for in the current study nor any previous studies.

In conclusion, using 10-year follow-up data from the large-scale, population-based 45 and Up Study, we found reduced risk of cataract surgery among participants who reported more outdoor time. This finding should be interpreted within the context of the many people who use sun protection in Australia. We found that subjects who tan easily had lower risk, suggesting a potential role between melanin and cataract. The reasons for this finding remain unclear and should be studied further.
